# Systematic immunohistochemical screening for mismatch repair and ERCC1 gene expression from colorectal cancers in China: Clinicopathological characteristics and effects on survival

**DOI:** 10.1371/journal.pone.0181615

**Published:** 2017-08-02

**Authors:** Pan Li, Zhitao Xiao, Todd A. Braciak, Qingjian Ou, Gong Chen, Fuat S. Oduncu

**Affiliations:** 1 Department of Hematology and Oncology, Medizinische Klinik und Poliklinik IV, Ludwig Maximilians University, Munich, Germany; 2 Department of Colorectal Surgery, State Key Laboratory of Oncology in South China, Sun Yat-sen University Cancer Center, Guangzhou, China; University of North Carolina at Chapel Hill School of Medicine, UNITED STATES

## Abstract

**Background:**

We performed a systematic screening of colorectal cancer (CRC) tissues to investigate whether mismatch repair (MMR) status and ERCC1 protein expression could be predictive of clinical outcomes for these patients following the recommendation of The Evaluation of Genomic Applications in Practice of Prevention (EGAPP).

**Methods:**

The expression of four MMR genes and ERCC1 were assessed by immunohistochemistry (IHC) from cancer tissue samples of 2233 consecutive CRC patients.

**Results:**

We observed that most CRC patients with a proficient MMR (pMMR) status tended to have simultaneous ERCC1 protein expression (*P*< 0.001). Stage III CRC patients with deficient MMR (dMMR) had higher prognoses than the same stage patients with pMMR (DFS: 74% vs 65%, *P* = 0.04; OS: 79% vs 69%, *P* = 0.04). Here, dMMR is also associated with poorer survival for stage II patients after chemotherapy (DFS: 66% vs 78%, *P* = 0.04). Stage II and III patients that were shown to express ERCC1 protein had higher DFS and OS than those that were deficient in expression (stage II, DFS: 83% vs 70%, *P* = 0.006; OS 85% vs 73%, *P* = 0.02. Stage III, DFS: 67% vs56%, *P* = 0.03; OS: 71% vs 57%, *P* = 0.04).

**Conclusions:**

Our results indicate that dMMR appeared to predictive of a survival benefit for stage III CRC patients. We also found the determination of ERCC1 expression to be useful for predicting DFS or OS for stage II and III CRC patients. In addition, the expression of MMR genes and ERCC1 showed a significant relationship.

## Introduction

In 2009 the Evaluation of Genomic Applications in Practice of Prevention (EGAPP) recommended screening MMR status for all newly diagnosed patients with colorectal cancer [1]. MMR corrects mismatched nucleotides and insertion-deletion loops (IDLs) in DNA caused by polymerase errors, chemical modifications, and recombination between heterologous DNA sequences [2]. MMR proteins also act as sensors to activate DNA damage checkpoints in response to alkylating agents and other DNA-damaging agents [3]. Thus, changes in MMR status could have significant impact on cancer onset and progression including CRC.

CRCs with dMMR have distinct clinical and pathological features that commonly include proximal colon predominance, poor differentiation and increased numbers of lymph nodes with metastases[4]. MMR status is increasingly used to guide clinical management. Stage II patients with dMMR have a better prognosis and may actually be harmed by 5-FU treatment [5]. Multiple studies have shown that CRCs with dMMRhave a better stage-adjusted survival compared with pMMR cancers. However, these data are largely from retrospective studies or focused on Lynch syndrome [6,7,8]. Molecular studies comparing CRCs with early and advanced stages have been rare.

Another pathway of DNA repair to operate on specific types of damaged DNA is the nucleotide-excision repair (NER) pathway [9]. ERCC1 is a key molecule in the NER pathway, which is responsible for repairing DNA adducts induced by platinum drugs [10,11]. It was reported that the ERCC1 expression was predictive for the sensitivity of oxaliplatin in colorectal cell lines [12]. Based on this evidence, several clinical studies have been conducted with the aim of relating ERCC1 tumor levels with response to oxaliplatin, but these results are still controversial. Moreover, all of these data were generated in the metastatic setting [13,14,15]. Although ERCC1 has a role as a prognostic marker in non–small-cell lung cancer (NSCLC)[16], for patients with CRC, the definite prognostic value of ERCC1 expression has not been established yet. And there is no study to explore the relationship between MMR status and ERCC1 expression.

Following the recommendations by EGAPP, systematic IHC screening for microsatellite instability of patients operated to remove their primary tumors for CRC has been assessed in our institute since 2011. At the same time, our group initiated testing for ERCC1 expression, in particular with immunohistochemistry as part of a standard fast routine test. For this report, we took advantage of this available data to analyze the detection rate, relationship, prognostic and predictive significance of MMR status and ERCC1 expression tested for all stages of CRC disease in China.

## Patients and methods

### Patients

The ethics committee of Sun Yat-sen University Cancer Center approved this study and informed consent for all patients was obtained at the beginning of the study. A total of 4500 histologically confirmed CRC patients were recruited after operation from Sun Yat-sen University cancer center between May 2011 and May 2016. All patients were of chinese origin. The clinical and family history of each of these patients was also reviewed. In our study, we analyzed the MMR status in sporadic CRC, while the dMMR frequency tends to be high in CRC patients with family history, especially for CRC patients with Lynch syndrome (LS). We also excluded patients with severe complications, multiprimary cancer and death not due to tumor-related reasons, in order to focus on the CRC-related survival analysis. Finally, 2233 cases were selected for analysis after application of strict exclusion criteria as outlined: age less than 18 years and older than 85 years (261 cases), severe complication (62 cases), multiprimary cancer, synchronous and metachronous CRC (135 cases), family history (first-degree and second-degree relatives had any kind of cancer) (258 cases), familial adenomatous polyposis (51 cases), incomplete follow-up records (1747 cases), death not due to tumor-related reason (47) were not included in the study. The primary tumor site was categorized as either proximal colon if the tumor was located above the splenic flexure or distal colon if it was located at or below the splenic flexure and rectum. The median follow-up for the surviving patients used in this study was 4.3 years. The patients’ information can be seen in the supporting information (S1 Data).

### Treatment and follow-up

Stage I (T1–2 N0) and stage II (T3–4 N0) CRC patients without high-risk clinical features (e.g. T4 stage, bowel perforation or clinical bowel obstruction, inadequate lymph node sampling, poorly differentiated histology) were treated with radical surgery or endoscopic removal of the tumor alone. Stage II (T3–4 N0) CRC patients with high-risk clinical features were recommended to receive XELODA/mFOLFOX/XELOX regimen treatments. Stage III (Tx N1–2) patients were to receive radical surgery and 12 cycles of adjuvant mFOLFOX/XELOX regimen treatment within a 6-month period. All stage IV (Tx Nx M1) patients received palliative surgery or radical surgery. The first-line treatment for Stage IV CRC was the mFOLFOX/FOLFIRI regimen. Eighty-nine patients with rectal cancer also received neo-chemoradiotherapy. Responses were evaluated in accordance with the RECIST guidelines. After surgery, tumor recurrence was detected by physical examination, serum carcinoembryonic antigen (CEA) assay, and abdominal and thoracic imaging monitoring analyzed every 3–6 months for the first 3 years, every 6 months for the following 2 years and then once annually for those patients surviving beyond the 5 year time point. The duration of follow-up was defined as the time between surgery and disease recurrence, death or last hospital contact (scheduled follow-up or telephone contact). The cutoff date for this analysis was May 2016.

### Screening

**Fig 1:**
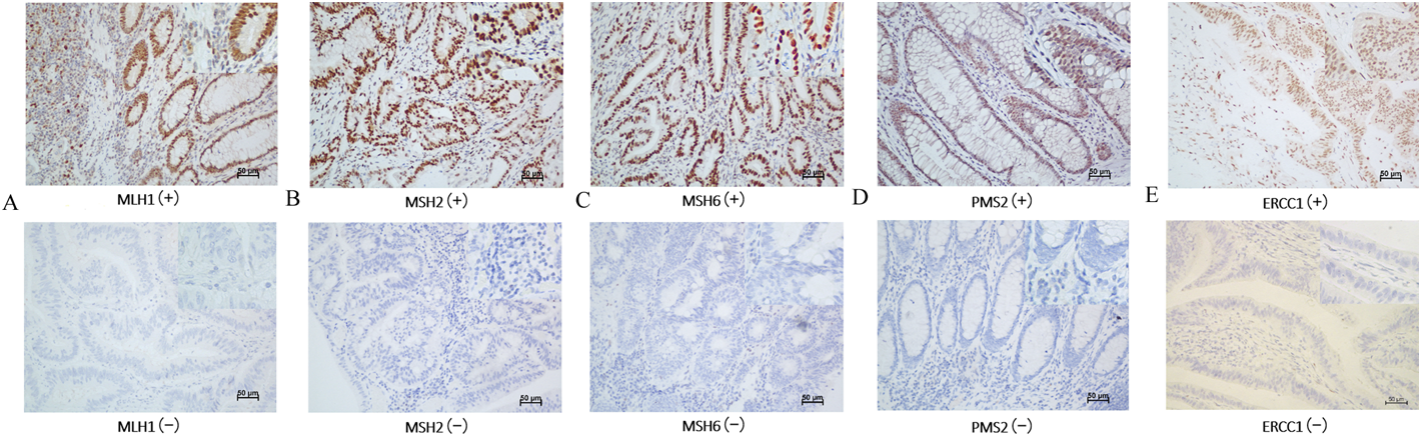
Illustrative immunostainings. A: MLH1(+) and (-); B:MSH2 (+) and (-); C: MSH6 (+) and (-); D: PMS2 (+) and (-); E: ERCC1 (+) and (-).

Blocks of formalin-fixed, paraffin-embedded adenocarcinoma tissue comprising an area of normal colorectal mucosa adjacent to the tumor were selected in each case. Cases with complete nuclear loss of expression in invasive tumor cells with retained expression in inflammatory cells and/or adjacent normal tissue as positive controls were considered MMR deficient or ERCC1 negative expression. Staining was performed using the following primary antibodies: mouse anti-human MLH1 (dilution 1:50, clone OTI1C1, zhongshan jiqiao, Beijing), rabbit anti-human MSH2 (dilution 1:200, clone ZA0622, zhongshan jiqiao, Beijing, mouse anti-human MSH6 (dilution 1:100, clone OTI5D1, zhongshan jiqiao, Beijing), mouse anti-human PMS2 (dilution 1:50, clone OTI2G5, zhongshan jiqiao, Beijing), and mouse anti-human ERCC1 (dilution 1:200, clone OTI1A3, zhongshan jiqiao, Beijing). Whole tissue sections were read separately by two pathologists blinded to the patients’ clinical characteristics. Discordant cases were reviewed by a supplementary pathologist to reach a consensus. Illustrative immunostainings were showed in Fig 1.

### Statistical analyses

Data were described as frequencies (percentages). Differences in distributions between the variables examined were assessed with the Χ^2^ or the Fisher’s exact test. The primary end point was DFS, defined as the time between the date of surgery and the first event (local or distant disease recurrence) or progression-free survival (PFS), calculated from the start of surgery until clinical or radiological progression. Patients who were alive and relapse free at the last contact were censored at the last follow-up date. Overall survival was defined as the time elapsed from the date of surgery until tumor-induced death. Surviving patients were censored on the last follow-up date. Median follow-up and the 95% CI were calculated using the reverse Kaplan–Meier method. Survival curve was estimated with the Kaplan–Meier method and compared using the log-rank test. Univariate and multivariable Cox proportional hazards models were used to explore the association of MMR status, ERCC1 expression, location, age, stage, differentiation grade and gender. The score and likelihood ratio test *P* values were used to test the statistical significance of each covariate in the univariate and multivariable Cox models, respectively. All statistical tests were two-sided, and *P* values less than or equal to 0.05 were considered statistically significant. Statistical analyses were performed using SPSS software.

## Results

Of the 2233 patients evaluated, 232 were found to have dMMR with an overall prevalence of 10.4%. 208 were found to have negative ERCC1 expression with prevalence of 9.3%. Detailed clinicopathological information for all patients is shown in Table 1.

**Table 1:** Clinicopathological information for all patients.

Characteristic	MMR status (n/%)		*P* value	ERCC1 expression (n/%)		*P* value
	dMMR (232/10.4)	pMMR (2001/89.6)		negative (208/9.3)	positive (2025/90.7)	
Gender			0.031			0.613
Male Female	152 (6.8) 80 (3.6)	1164(52.1) 837(37.5)		126 (5.6) 82 (3.7)	1190 (53.3) 835 (37.4)	
Age			0.001			0.872
20–39 years 40–59 years 60–85 years	26 (1.2) 125 (5.6) 81(3.6)	162 (7.3) 899 (40.3) 940(42.1)		13 (0.6) 103 (4.6) 92 (4.1)	175 (7.8) 921 (41.2) 929 (41.6)	
Pathology			0.002			0.194
G1 G2 G3 Mucinous Signet-ring	21 (0.9) 181 (8.1) 2 (0.1) 26 (1.2) 2 (0.1)	83 (3.7) 1821 (81.5) 16 (0.7) 69 (3.1) 12 (0.5)		6 (0.3) 188 (8.4) 1 (0.0) 11 (0.5) 2 (0.1)	98 (4.4) 1814 (81.2) 17 (0.8) 84 (3.8) 12 (0.5)	
Stage			<0.001			0.491
I IIA IIB IIC IIIA IIIB IIIC IVA IVB	30 (1.3) 101 (4.5) 18 (0.8) 8 (0.4) 6 (0.3) 37 (1.7) 11 (0.5) 14 (0.6) 7 (0.3)	301 (13.5) 509 (22.8) 207 (9.3) 34 (1.5) 49 (2.2) 415 (18.6) 81 (3.6) 251 (11.2) 154 (6.9)		27 (1.2) 65 (2.9) 24 (1.1) 8 (0.4) 4 (0.2) 33 (1.5) 7 (0.3) 20 (0.9) 20 (0.9)	304 (13.6) 545 (24.4) 201 (9.0) 34 (1.5) 51 (2.3) 419 (18.8) 85 (3.8) 245 (11.0) 141 (6.3)	
Location			<0.001			0.855
Right colon Left colon Rectum	117 (5.2) 55 (2.5) 60 (2.7)	404 (18.1) 685 (30.7) 912 (40.8)		50 (2.2) 64 (2.9) 94 (4.2)	471 (21.1) 676 (30.3) 878 (39.3)	
MMR status			<0.001			<0.001
dMMR pMMR	232 (10.4) 0 (0.0)	0 (0.0) 2001 (89.6)		48 (2.1) 160 (7.2)	184 (8.2) 1841 (82.4)	
ERCC1			<0.001			<0.001
Negative positive	48 (2.1) 184 (8.2)	160 (7.2) 1841 (82.4)		208 (9.3) 0 (0.0)	0 (0.0) 2025 (90.7)	
Metastasis			0.006			0.028
Yes No	60 (2.7) 172 (7.7)	699 (31.3) 1302 (58.3)		85 (3.8) 123 (5.5)	674 (30.2) 1351 (60.5)	
Live			0.008			0.019
Yes No	178 (8.0) 54 (2.4)	1366 (61.2) 635 (28.4)		129 (5.8) 79 (3.5)	1415 (63.4) 610 (27.3)	

### Frequency of MMR status and expression of ERCC

A total of 2001 (89.6%) CRC specimens showed retained expression of MLH1, MSH2, MSH6 and PMS2 in tumor cells. In comparison, loss of expression in at least one of the four MMR genes occurred only in 232 of 2233 patients (10.4%). The distribution of loss of expression of the MMR genes was as following: combined MLH1/PMS2 loss (n = 79; 34.1%), combined MSH2/MSH6 loss (n = 33; 14.2%), combined MLH1/MSH2/MSH6/PMS2 loss (n = 7; 3.0%), combined MLH1/MSH6 loss (n = 4; 1.7%), combined MLH1/MSH6/PMS2 loss (n = 5; 2.2%), combined MLH1/MSH2/PMS2 loss (n = 5; 2.2%), isolated MLH1 loss (n = 17; 7.3%), isolated PMS2 loss (n = 12; 5.2%), isolated MSH2 loss (n = 16; 6.9%), isolated MSH6 loss (n = 54; 23.3%). We stratified the clinical characteristics of the study population by MMR status. The dMMR vs pMMR CRC were more likely to be stage IIA (16.6%) vs others (stage I: 9.1%, stage IIB: 8.0%, stage IIC: 19.0%, stage IIIA: 10.9%, stage IIIB: 8.4%, stage IIIC: 11.9%, stage IVA: 5.3%, stage IVB: 4.3%, *P* <0.001), right colon (22.5%) vs left colon (7.4%) and rectum (6.2%) (*P <* 0.001), from men (11.6%) vs women (8.7%) (*P* = 0.031), and poor or undifferentiated (23.6%) vs well or moderate differentiation (9.6%) (*P* < 0.001), young age (20–39 years old, 13.8%) vs old age (40–59 years, 12.2%; 60–85 years old, 8.3%; *P* = 0.001), ERCC1 negative (23.1%) vs ERCC1 positive (9.1%) (*P*< 0.001). The multi-analysis results are shown in Table 2. In all, 208 cases (9.3%) had negative ERCC1 tumors and 2015 cases (90.7%) had positive tumors. Gender, age, location, stage and pathological differentiation showed no statistic difference in univariate Cox analysis (*P*>0.05) for ERCC1 expression.

**Table 2:** Multi-variate analysis of disease-free survival and overall survival.

		95% CI of DFS				95% CI of OS		
Variable	HR	Lower	Upper	*P* value	HR	Lower	Upper	*P* value
Age	2.24	2.16	2.34	0.001	2.38	2.36	2.42	<0.001
Gender	1.35	1.28	1.41	0.031	1.41	1.39	1.44	0.032
Stage	3.54	3.19	3.89	<0.001	3.19	3.23	3.85	<0.001
Location	2.25	2.22	2.28	<0.001	1.75	1.66	1.86	0.001
Grade	2.17	2.10	2.14	0.002	2.23	2.07	2.26	0.001
ERCC1	0.79	0.76	0.83	<0.001	0.92	0.91	0.93	<0.001

### Assessment of MMR as prognostic marker

**Fig 2:**
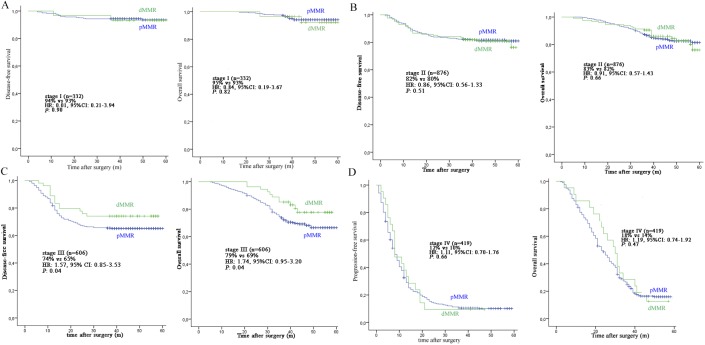
The survival plots of MMR status. A: stage I patients; B: stage II patients; C: stage III patients; D: stage IV patients.

MMR status provided prognostic information in CRC patients. Patients with stage III CRC with dMMR tumors showed a statistically significant improvement in DFS (74%) and OS (79%), compared with patients with pMMR tumors (DFS 65%; HR: 1.57, 95% CI: 0.85–3.53, *P* = 0.04 and OS 69%; HR: 1.74, 95% CI: 0.95–3.20, *P* = 0.04). This was in contrast to the results of analysis obtained for stage I patients where no difference in the 3-year DFS (93%) and OS (93%) was found between dMMR versus patients with pMMR (DFS 94%; HR: 0.01, 95% CI: 0.21–3.94, *P* = 0.90 and OS 95%; HR: 0.84, 95% CI: 0.19–3.67, *P* = 0.82). For stage II patients, we also found no difference in the 3-year DFS (82%) and OS (83%) between the dMMR group versus the pMMR group (DFS 80%; HR: 0.86, 95% CI: 0.56–1.33, *P* = 0.51 and OS 82%; HR: 0.91, 95% CI: 0.57–1.43, *P* = 0.66). Finally, we found no difference in the 3-year DFS (12%) and OS (18%) for stage IV patients between the dMMR group versus the pMMR group (DFS 10%; HR: 1.11, 95% CI: 0.70–1.76, *P* = 0.66 and OS 14%; HR: 1.19, 95% CI: 0.74–1.92, *P* = 0.47). The survival plots of MMR status are shown in Fig 2.

### Assessment of MMR as Predictive Marker for Stage II CRC

**Fig 3:**
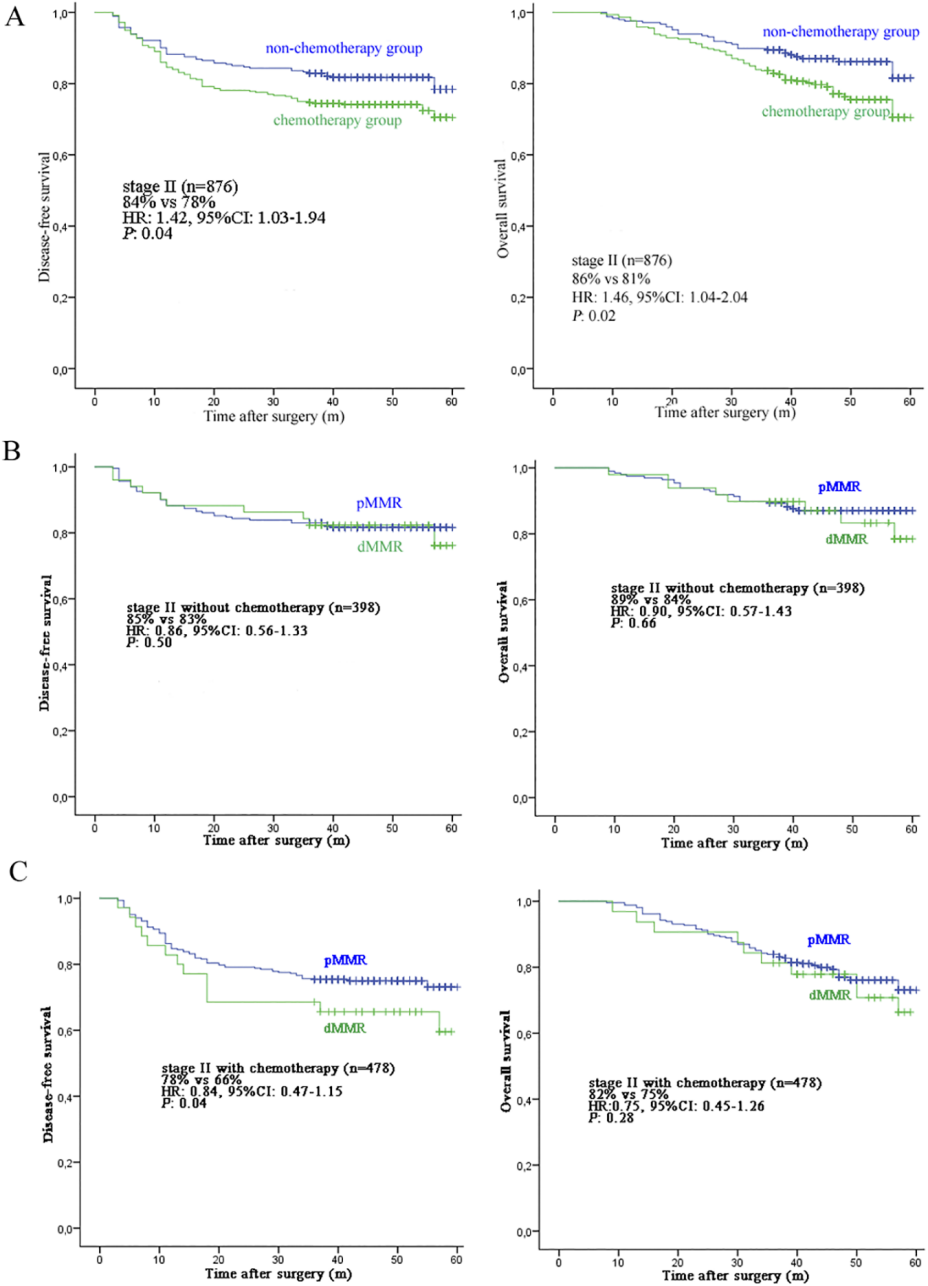
The survival plots of chemotherapy and MMR status. A: all stage II patients; B: stage II patients without chemotherapy; C: stage II patients with chemotherapy.

The 3-year DFS of stage II patients with chemotherapy and without chemotherapy was 78% and 84%, respectively (HR: 1.42, 95% CI: 1.03–1.94, *P* = 0.03). The OS rate between chemotherapy group and non-chemotherapy group was 81% and 86%, respectively (HR: 1.46, 95% CI: 1.04–2.04, *P* = 0.02), confirming that addition of chemotherapy to treatments for stage II CRC damages the prognosis within this setting of patients. However, the 3-year DFS was significantly lower in stage II patients with chemotherapy with dMMR tumors (66%) than in the same group patients with pMMR tumors (78%, HR: 0.84, 95%CI: 0.47–1.15, *P* = 0.04). Despite this finding for DFS, the OS showed no statistical difference in stage II patients with chemotherapy with dMMR tumor (75%) and in the same group of patients with pMMR tumors (82%, HR: 0.75, 95%CI: 0.45–1.26, *P* = 0.28). Also, no difference in the 3-year DFS (85%) and OS (89%) for stage II patients without chemotherapy between the dMMR group versus the pMMR group (DFS 83%; HR: 0.86, 95% CI: 0.56–1.33, *P* = 0.50 and OS 84%; HR: 0.90, 95% CI: 0.57–1.43, *P* = 0.66). The survival plots of chemotherapy and MMR status are shown in Fig 3.

### Assessment of ERCC1 as prognostic marker

**Fig 4:**
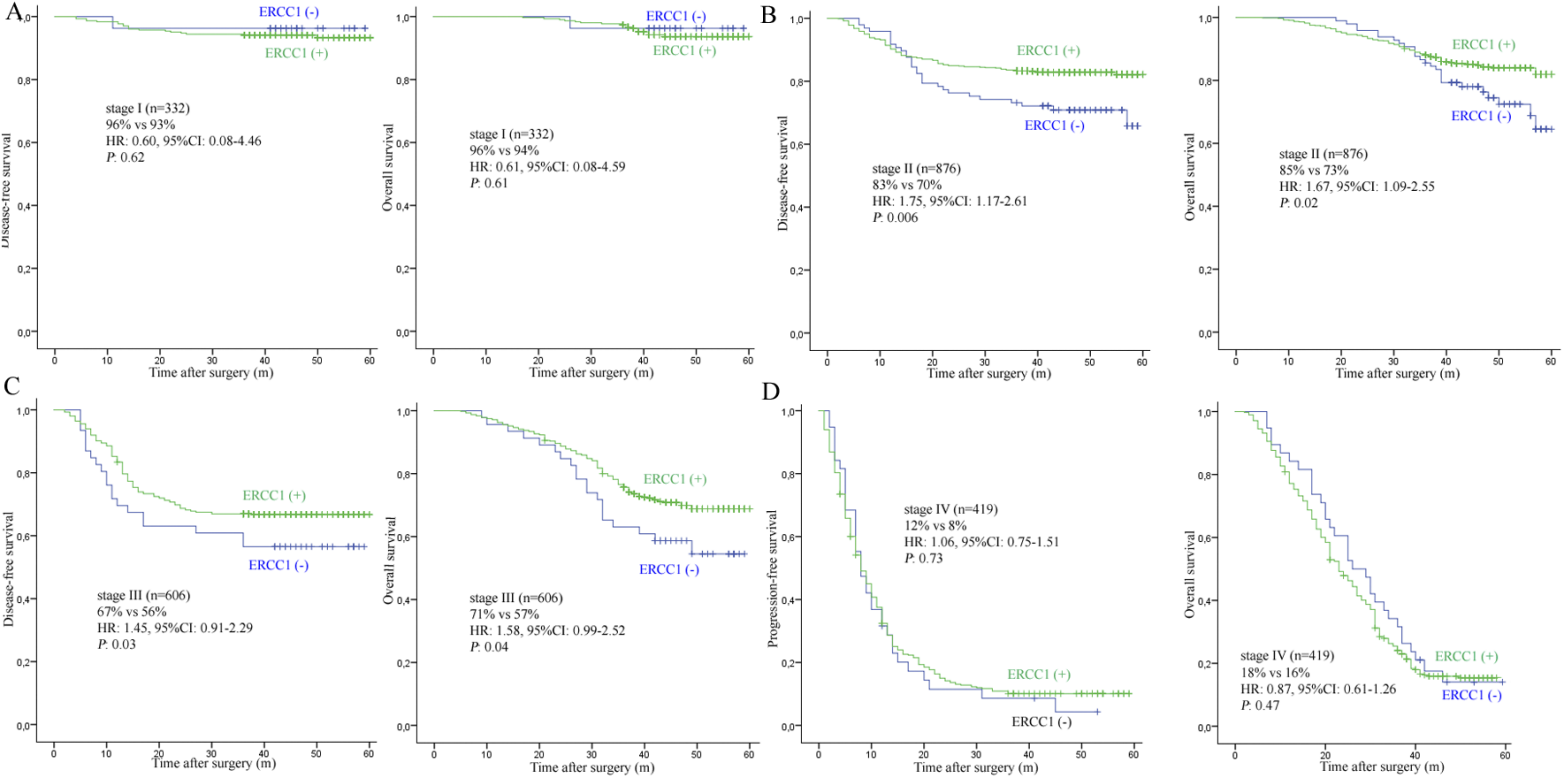
The survival plots of ERCC1 expression. A: stage I patients; B: stage II patients; C: stage III patients; D: stage IV patients.

The group with negative ERCC1 expression showed no statistically significant improvement of 3-year DFS or OS in stage I patients (DFS: 96% vs 93%, HR: 0.60, 95%CI: 0.08–4.46, *P* = 0.62; OS: 96% vs 94%, HR: 0.61, 95%CI: 0.08–4.59, *P* = 0.61). However, in stage II patients, the 3-year DFS and OS in ERCC1 positive group was statistically significantly higher than in ERCC1 negative group (DFS: 83% vs 70%, HR:1.75, 95%CI: 1.17–2.61, *P* = 0.006; OS: 85% vs 73%, HR:1.67, 95%CI: 1.09–2.55, *P* = 0.02). In addition, patients with stage III CRC with positive ERCC1 tumors also showed a statistically significant improvement in DFS (67%) and OS (71%), compared to patients with negative ERCC1 tumors (DFS 56%; HR: 1.45, 95% CI: 0.91–2.29, *P* = 0.03 and OS 57%; HR: 1.58, 95% CI: 0.9–2.52, *P* = 0.04). Finally, we found no difference in the 3-year DFS (12%) and OS (16%) for stage IV patients between the positive ERCC1 group versus the negative group (DFS 8%; HR: 1.06, 95% CI: 0.75–1.51, *P* = 0.73 and OS 18%; HR: 0.87, 95% CI: 0.61–1.26, *P* = 0.47). These associations toward better survival for ERCC1 expression for stage II and III patients versus stage I or IV patients indicate that ERCC1 might be a useful marker for analysis of CRC disease. The survival plots of ERCC1 expression are shown in Fig 4.

## Discussion

This current study presents a large dataset exploring a role for tumor MMR status and ERCC1 expression with respect to prevalence of CRC and disease outcome in a population of chinese patients (n = 2233).

Tumors with dMMR usually show complete loss of expression of one or more MMR protein. Here, all four MMR genes (MLH1, MSH2, MSH6 and PMS2) were analyzed by IHC for detection. The most frequent MMR gene expression pattern found was the concurrent loss of MLH1 and PMS2, which accounted for 34.1% of all CRC cases studied in our analysis. The second most common pattern found was the isolated loss of the MSH6 gene that accounted for 23.3% of the CRC tumors analyzed and is similar to the result of previous related studies regarding detection of MMR gene mutations[17,18]. In our study, we excluded patients with familial cancer history, which included the high-penetrant Lynch families (those with MLH1 and MSH2 germline alternations), the remaining patients with lesser penetrant LS (those with MSH6 and PMS2 germline mutations) can be the reason for the high percentage of MSH6 loss in our study.

In our study, the incidence rate of dMMR tumor was only 10.4%, which is lower than the published incidence rates (15–25%) of dMMR found for African-American CRCs[19,20] or 15% found for mixed race populations[21]. We found the MMR mutation frequency was significantly lower in our study indicating that a significant proportion of CRC in China may actually follow tumorigenesis pathways distinct from the dMMR CRC progression sequence. Clearly, this possible heterogeneity could also have implications for CRC prognosis and the clinical management of disease.

We found that the association between a favorable outcome and dMMR status showed no statistical significance for DFS and OS of the stage II cohort of patients. However, 5-FU chemotherapy tended towards poorer prognosis for stage II patients with dMMR, a result that is discordant with other previous studies [7,22]. Compared to stage III CRCs, more stage II CRCs were with dMMR, especially the stage IIA tumors that had a higher dMMR rate of 16.6%. In our study, MMR status appeared to act as an independent prognostic biomarker for DFS in patients with stage III colon cancer that had received adjuvant FOLFOX chemotherapy a result that is consistent with other recent studies[6,23]. In metastatic CRC (mCRC), we found that the prevalence of dMMR was low (4.9%). This finding supports the hypothesis that dMMR tumors have a reduced metastatic potential. The low prevalence of dMMR in mCRC could be explained by the reduced potential of stage I-III dMMR tumors to metastasize [24]. However the underlying mechanisms of this low metastatic potential are yet to be elucidated. In terms of the prognostic value of dMMR in mCRC, our data showed no statistical difference between dMMR versus the pMMR cohort of patients.

Although ERCC1 has a role as a prognostic marker in NSCLC[16], only a few studies have evaluated its role as a prognostic marker in colorectal cancer and most of the previous data were generated in the metastatic setting of cancer[13,14,15]. In this current study, we have found that Chinese CRC patients with ERCC1 expression have a significantly better prognosis than the negative cohort group with stage II and III disease. However, our data showed no statistical difference between ERCC1 positive and negative cohorts of patients that had stage I or IV disease indicating other factors are also involved in the pathogenesis of the disease. While a report on NSCLC patients found that ERCC1 expression was significantly lower in female than male cancer patients, our study did not find any significant differences in sex, tumor locations, pathological differentiation, substages or ages for ERCC1 expression.

Finally, our large dataset showed that patients with pMMR status tended to also have positive ERCC1 expression, suggesting a collaboration of these two DNA repair pathways in maintaining cell integrity and normalcy. MMR proteins are responsible for correcting mismatched nucleotides and insertion-deletion loops (IDLs) in DNA caused by polymerase errors, chemical modifications, and recombination between heterologous DNA sequences[7], while ERCC1 is a key molecule in the nucleotide excision repair (NER) pathway, which is responsible for repairing DNA adducts induced by platinum drugs[14,15]. The underlying mechanisms of these potential interactions between these DNA repair proteins still needs to be elucidated to gain a better understanding of CRC pathogenesis and its prognosis.

## Conclusion

Our study is the first big dataset research following the recommendation of “The Evaluation of Genomic Applications in Practice of Prevention” (EGAPP) since 2011. Our results provide cutting-edge insights for the evaluation of the significance of MMR status for the prognosis and treatment of CRC. Moreover, it is also the first persuasive study showing the correlation between MMR status and ERCC1 expression. Hence, further research about the correlation of the pathway of mismatch repair and nucleotide-excision repair is needed.

## Supporting information

S1 Data (XLSX)

## References

[1] (2009) Recommendations from the EGAPP Working Group: genetic testing strategies in newly diagnosed individuals with colorectal cancer aimed at reducing morbidity and mortality from Lynch syndrome in relatives. Genet Med 11: 35–41. doi: 10.1097/GIM.0b013e31818fa2ff 19125126 10.1097/GIM.0b013e31818fa2ff PMC2743612

[2] Kolodner RD, Marsischky GT (1999) Eukaryotic DNA mismatch repair. Curr Opin Genet Dev 9: 89–96. 10072354 10.1016/s0959-437x(99)80013-6

[3] Jiricny J (2006) The multifaceted mismatch-repair system. Nat Rev Mol Cell Biol 7: 335–346. doi: 10.1038/nrm1907 16612326 10.1038/nrm1907

[4] Jass JR, Do KA, Simms LA, Iino H, Wynter C, Pillay SP, et al (1998) Morphology of sporadic colorectal cancer with DNA replication errors. Gut 42: 673–679. 9659163 10.1136/gut.42.5.673 PMC1727100

[5] Boland CR, Goel A (2010) Microsatellite instability in colorectal cancer. Gastroenterology 138: 2073–2087. doi: 10.1053/j.gastro.2009.12.064 20420947 10.1053/j.gastro.2009.12.064 PMC3037515

[6] Zaanan A, Bachet JB, Andre T, Sinicrope FA (2014) Prognostic Impact of Deficient DNA Mismatch Repair and Mutations in KRAS, and BRAFV600E in Patients with Lymph Node-Positive Colon Cancer. Curr Colorectal Cancer Rep 10: 346–353. doi: 10.1007/s11888-014-0237-2 25386108 10.1007/s11888-014-0237-2 PMC4224319

[7] Lanza G, Gafa R, Santini A, Maestri I, Guerzoni L, Cavazzini L. (2006) Immunohistochemical test for MLH1 and MSH2 expression predicts clinical outcome in stage II and III colorectal cancer patients. J Clin Oncol 24: 2359–2367. doi: 10.1200/JCO.2005.03.2433 16710035 10.1200/JCO.2005.03.2433

[8] Jemal A, Siegel R, Ward E, Hao Y, Xu J, Thun MJ. (2009) Cancer statistics, 2009. CA Cancer J Clin 59: 225–249. doi: 10.3322/caac.20006 19474385 10.3322/caac.20006

[9] Goode EL, Ulrich CM, Potter JD (2002) Polymorphisms in DNA repair genes and associations with cancer risk. Cancer Epidemiol Biomarkers Prev 11: 1513–1530. 12496039

[10] Youn CK, Kim MH, Cho HJ, Kim HB, Chang IY, Chung MH, et al (2004) Oncogenic H-Ras up-regulates expression of ERCC1 to protect cells from platinum-based anticancer agents. Cancer Res 64: 4849–4857. doi: 10.1158/0008-5472.CAN-04-0348 15256455 10.1158/0008-5472.CAN-04-0348

[11] Arnould S, Hennebelle I, Canal P, Bugat R, Guichard S (2003) Cellular determinants of oxaliplatin sensitivity in colon cancer cell lines. Eur J Cancer 39: 112–119. 12504667 10.1016/s0959-8049(02)00411-2

[12] Chang IY, Kim MH, Kim HB, Lee DY, Kim SH, You HJ. (2005) Small interfering RNA-induced suppression of ERCC1 enhances sensitivity of human cancer cells to cisplatin. Biochem Biophys Res Commun 327: 225–233. doi: 10.1016/j.bbrc.2004.12.008 15629453 10.1016/j.bbrc.2004.12.008

[13] Shirota Y, Stoehlmacher J, Brabender J, Xiong YP, Uetake H, Danenberg KD, et al (2001) ERCC1 and thymidylate synthase mRNA levels predict survival for colorectal cancer patients receiving combination oxaliplatin and fluorouracil chemotherapy. J Clin Oncol 19: 4298–4304. doi: 10.1200/JCO.2001.19.23.4298 11731512 10.1200/JCO.2001.19.23.4298

[14] Viguier J, Boige V, Miquel C, Pocard M, Giraudeau B, Sabourin JC, et al (2005) ERCC1 codon 118 polymorphism is a predictive factor for the tumor response to oxaliplatin/5-fluorouracil combination chemotherapy in patients with advanced colorectal cancer. Clin Cancer Res 11: 6212–6217. doi: 10.1158/1078-0432.CCR-04-2216 16144923 10.1158/1078-0432.CCR-04-2216

[15] Nishina T, Takano Y, Denda T, Yasui H, Takeda K, Ura T, et al (2013) A phase II clinical study of mFOLFOX6 plus bevacizumab as first-line therapy for Japanese advanced/recurrent colorectal cancer patients. Jpn J Clin Oncol 43: 1080–1086. doi: 10.1093/jjco/hyt127 23999770 10.1093/jjco/hyt127 PMC3814899

[16] Olaussen KA, Dunant A, Fouret P, Brambilla E, Andre F, Haddad V, et al (2006) DNA repair by ERCC1 in non-small-cell lung cancer and cisplatin-based adjuvant chemotherapy. N Engl J Med 355: 983–991. doi: 10.1056/NEJMoa060570 16957145 10.1056/NEJMoa060570

[17] Southey MC, Jenkins MA, Mead L, Whitty J, Trivett M, Tesoriero AA, et al (2005) Use of molecular tumor characteristics to prioritize mismatch repair gene testing in early-onset colorectal cancer. J Clin Oncol 23: 6524–6532. doi: 10.1200/JCO.2005.04.671 16116158 10.1200/JCO.2005.04.671

[18] Hall G, Clarkson A, Shi A, Langford E, Leung H, Eckstein RP, et al (2010) Immunohistochemistry for PMS2 and MSH6 alone can replace a four antibody panel for mismatch repair deficiency screening in colorectal adenocarcinoma. Pathology 42: 409–413. doi: 10.3109/00313025.2010.493871 20632815 10.3109/00313025.2010.493871

[19] Sylvester BE, Huo D, Khramtsov A, Zhang J, Smalling RV, Olugbile S, et al (2012) Molecular analysis of colorectal tumors within a diverse patient cohort at a single institution. Clin Cancer Res 18: 350–359. doi: 10.1158/1078-0432.CCR-11-1397 22114137 10.1158/1078-0432.CCR-11-1397 PMC3272273

[20] Kumar K, Brim H, Giardiello F, Smoot DT, Nouraie M, Lee EL, et al (2009) Distinct BRAF (V600E) and KRAS mutations in high microsatellite instability sporadic colorectal cancer in African Americans. Clin Cancer Res 15: 1155–1161. doi: 10.1158/1078-0432.CCR-08-1029 19190129 10.1158/1078-0432.CCR-08-1029 PMC2713502

[21] Jenkins MA, Hayashi S, O'Shea AM, Burgart LJ, Smyrk TC, Shimizu D, et al (2007) Pathology features in Bethesda guidelines predict colorectal cancer microsatellite instability: a population-based study. Gastroenterology 133: 48–56. doi: 10.1053/j.gastro.2007.04.044 17631130 10.1053/j.gastro.2007.04.044 PMC2933045

[22] Bertagnolli MM, Redston M, Compton CC, Niedzwiecki D, Mayer RJ, Goldberg RM, et al (2011) Microsatellite instability and loss of heterozygosity at chromosomal location 18q: prospective evaluation of biomarkers for stages II and III colon cancer—a study of CALGB 9581 and 89803. J Clin Oncol 29: 3153–3162. doi: 10.1200/JCO.2010.33.0092 21747089 10.1200/JCO.2010.33.0092 PMC3157981

[23] Zaanan A, Flejou JF, Emile JF, Des GG, Cuilliere-Dartigues P, Malka D, et al (2011) Defective mismatch repair status as a prognostic biomarker of disease-free survival in stage III colon cancer patients treated with adjuvant FOLFOX chemotherapy. Clin Cancer Res 17: 7470–7478. doi: 10.1158/1078-0432.CCR-11-1048 21998335 10.1158/1078-0432.CCR-11-1048

[24] Malesci A, Laghi L, Bianchi P, Delconte G, Randolph A, Torri V, et al (2007) Reduced likelihood of metastases in patients with microsatellite-unstable colorectal cancer. Clin Cancer Res 13: 3831–3839. doi: 10.1158/1078-0432.CCR-07-0366 17606714 10.1158/1078-0432.CCR-07-0366

